# Colony and Single Cell Level Analysis of the Heterogeneous Response of *Cryptococcus neoformans* to Fluconazole

**DOI:** 10.3389/fcimb.2018.00203

**Published:** 2018-06-19

**Authors:** Sophie Altamirano, Charles Simmons, Lukasz Kozubowski

**Affiliations:** Department of Genetics and Biochemistry, Clemson University, Clemson, SC, United States

**Keywords:** *Cryptococcus neoformans*, fluconazole, drug susceptibility, drug resistance, phenotypic heterogeneity

## Abstract

*Cryptococcus neoformans* is a human fungal pathogen that can cause fatal meningitis in immunocompromised individuals. Fluconazole (FLC) is a fungistatic drug administered to treat cryptococcosis. When exposed to the inhibitory concentration of FLC, *C. neoformans* exhibits heteroresistance where a small subpopulation of cells develops into FLC-resistant colonies. FLC-resistant cells are aneuploids with regard to specific beneficial chromosomal regions. Factors underlying the potential for only certain *C. neoformans* cells in a genetically isogenic population to become FLC-resistant are unknown. In this study, we systematically examine the heterogeneous response of *C. neoformans* to FLC at a colony and individual cell level. We find that the heterogeneity in response to FLC is reflected by variable diminishment of the ergosterol at the plasma membrane. A population of *C. neoformans* spread on a semi-solid medium displays two types of outcomes following FLC exposure. The first outcome is colonies consisting of non-resistant cells (survivors). The size of colonies consisting of survivors ranges from a few cells to visible colonies, which reflects intrinsic phenotypic heterogeneity of the *C. neoformans* population. The second outcome is FLC-resistant cells forming colonies of sizes significantly larger as compared to colonies made of survivors. We propose a model that describes how a distribution of these types of cellular responses within a population changes depending on FLC concentration and factors that influence the rate of cellular growth including temperature, media type, growth phase, and the age of cells. Our findings highlight a complex nature of the response to a fungistatic drug and provide insights that may help to optimize FLC therapy.

## Introduction

Single-cell clonal populations are made up of genetically homogeneous cells, but genetic identity does not necessarily translate to phenotypic similarity. Phenotypic heterogeneity has been shown to provide diversity among genetically clonal populations, allowing for adaptation to the environment without permanently locking the cells into a particular fate. It can be defined with respect to multiple aspects with various underlying physiological occurrences (Avery, 2006; Huang, 2009; Sharma et al., 2010; van Dijk et al., 2015). Of particular importance is the heterogeneous response of pathogens to drug treatment, especially in the context of the development of drug resistance as it constitutes a major barrier to effective therapy.

Drug therapies that aim at eliminating a certain cell type (i.e., microbial pathogens, cancer cells) often overlook the phenotypic heterogeneity that exists within the targeted cell population and its potential to promote survival during treatment. For example, one distinct challenge to antimicrobial therapy is the occurrence of “persister” subpopulations of slow growing cells, which contribute to recurrent infections (Cohen et al., 2013). Persisters are thought to remain throughout infection; they do not make up the population of truly resistant cells, but may contribute to the formation of resistant cells through the ability to survive in the presence of the drug. Although more commonly studied in bacterial populations, the presence of persisters has been characterized in several eukaryotic pathogens and cancer cells (Cohen et al., 2013). An important question to consider when aiming at identifying a drug therapy that does not allow for resistance or recurrence is: Why are some genetically identical cells able to proliferate when challenged with a drug while others are inhibited? A better understanding of the intrinsic, phenotypic heterogeneity of cell populations is crucial to improve our current therapeutic approaches.

In this study, we addressed the intrinsic heterogeneity within the population of *Cryptococcus neoformans*, a human fungal pathogen, in the context of the response to an azole drug, fluconazole (FLC) (Perfect et al, 2010). FLC inhibits lanosterol 14α-demethylase, Erg11p, which is an essential enzyme for the synthesis of ergosterol, an important sterol present in the cellular membranes and enriched in the plasma membrane (Zhang et al., 2010). Previous studies have defined a phenomenon of heteroresistance in *C. neoformans* as an intrinsic ability to develop a small subpopulation of aneuploid, FLC-resistant cells, when exposed to the inhibitory concentrations of FLC (Sionov et al., 2009, 2010, 2013). Specific genes that confer resistance to FLC in *C. neoformans* are well-established (Selmecki et al., 2010; Kwon-Chung and Chang, 2012). On the other hand, mechanisms through which FLC potentially contributes to the development of resistance remain poorly characterized. Former studies have demonstrated that the response of *C. neoformans* to FLC is heterogeneous (Sionov et al., 2010, 2013). A plasticity of gene duplication patterns at the single colony level was observed, which suggested that the process of multiple chromosome duplication vary among individual cells (Sionov et al., 2010). These findings suggested that *C. neoformans* exhibits an inherent, non-genetic heterogeneity that influences the response to the drug. However, the nature of the heterogeneous response of *C. neoformans* to FLC has not been thoroughly investigated.

We characterized the response of *C. neoformans* to FLC using colony and single-cell level analyses. We provide evidence that individual cells in the population exhibit variable diminishment of ergosterol within the plasma membrane during the initial exposure to FLC. Although a causal relationship remains to be tested, the heterogeneity in ergosterol level is reflected by the variable sizes of colonies that arise on a semi-solid medium supplemented with FLC. The resulting colonies can be divided into two groups: colonies that consist of non-resistant cells (survivors) and colonies that contain primarily resistant cells. Growth conditions that promote higher growth rate, including high nutrient content or higher temperature, lead to a diminishment of size of the colonies consisting of survivors and a decrease in number of resistant colonies in the presence of FLC. Conversely, conditions that lead to a slower growth rate, including nutrient poor media, lower temperature, and stationary phase of growth result in a more successful proliferation of survivors in the presence of FLC. Consistent with these results, relatively young cells form smaller colonies upon FLC exposure, as compared to the remaining population. Analysis of cell morphology indicates that conditions that promote slower growth may lead to a delay in daughter cell separation, which may contribute to a better survival in the presence of FLC. Based on our data, we propose a model, which describes how FLC concentration and temperature modulate distribution of colonies that contain non-resistant and resistant *C. neoformans* cells. Our study provides insights that may help to improve *in vitro* drug susceptibility testing and augment treatments of cryptococcal infections.

## Materials and methods

### Growth conditions

Strains used in this study are *C. neoformans* var. *grubii* H99 (wild type, mating type α, obtained from the laboratory of Joseph Heitman, #4413), KN99a (wild type mating type **a**, obtained from the laboratory of Joseph Heitman, #3259; Nielsen et al., 2003) and a strain isogenic to H99, in which a sequence encoding fluorescent protein mCherry was introduced to replace the STOP codon of the gene encoding histone H4 (Sutradhar et al., 2015). Unless otherwise stated, cells were grown in liquid YPD overnight at 24°C and refreshed next day to 0.2 at 600 nm (OD_600_) before treatment. For FLC-treated cultures, FLC (Alfa Aesar, Haverhill, MA) was prepared to a final concentration from a 50 mg/ml stock in DMSO. For experiments at 30°C or 37°C, cells were pre-incubated in YPD overnight at 30 or 37°C.

### Flow cytometry

Cells were harvested before exceeding ~0.8 OD_600_, spun down, washed with sterile water, suspended in 100 μl distilled water, and fixed with 70% EtOH (in a dropwise manner while vortexing). Cells were then incubated at 24°C for 1 h and transferred to 4°C overnight. Next day, cells were washed with RNAse A buffer (0.2 M Tris pH 7.5, 20 mM EDTA), suspended in 100 μl of RNAse A buffer with 1 μl RNAse A (10 mg/ml), and incubated for 4 h at 37°C. Cells were then washed twice with 1 ml phosphate-buffered saline (PBS), suspended in 900 μl of PBS, and incubated at 4°C overnight. Cells were stained with propidium iodide (PI) by adding 100 μl of 0.005 mg/ml PI stock and incubated in the dark for 30 min. Immediately before analysis, cells were sonicated at 20% amplitude for 5 s to avoid clumping. For ploidy analysis, the PI fluorescence was collected from 10,000 cells using FL3 (488 nm laser) on BD Accuri C6 flow cytometer. Cell sorting was performed using Biorad S3E cell sorter. At least 500,000 cells were sorted into two fractions based on cells labeled or not labeled with FITC using FL1 (488 nm laser).

In order to separate young cells from the population, unbudded cells were selected via centrifugation. To this end, cells were grown in 200 ml for 2 days then pelleted and suspended in 50% sorbitol (1M) and 50% YPD. Cells were spun at 2,000 rpm for 5 min. The supernatant was transferred to a new tube and spun at 1,500 rpm for 5 min. Again, the supernatant was transferred to a new tube and spun at 4,000 rpm for 10 min. The morphology of the pelleted cells was then assessed under the microscope to ensure the majority of the population was unbudded. Cells were then biotinylated using EZ-Link Sulfo-NHS-LC-Biotin (ThermoScientific) and stained with fluorescein (FITC) (Sigma, St. Louis, MO) (Maxson et al., 2007). To this purpose, cells were washed three times with PBS and suspended to a density of 5 × 10^7^ cells/ml. Subsequently, 4 mg/ml of Sulfo-NHS-LC-Biotin reagent was added. Cells were incubated at 24°C for 30 min then washed three times with YPD. Biotinylated cells were released into YPD for an additional 4 h and then washed three times and stained with FITC (1:200) for 10 min. Cells were then washed and released into PBS to be sorted.

### Microscopy, imaging, and image processing

Bright field and fluorescence images were captured by AxioCam HRm monochromatic camera using the 100x EC-Plan-NEOFLUAR objective mounted on a Zeiss Axiovert 200 inverted microscope (Carl Zeiss, Thornwood, NY) interfaced with AxioVision Rel 4.8 software (Carl Zeiss, Thornwood, NY). Colony dissection was performed using SporePlay tetrad dissection microscope (Singer Instruments, UK). Microcolonies were imaged by the PixeLINK color camera (PixeLINK, Ottawa, Canada) using the LWD 40x/0.45 objective mounted on the SporePlay dissection microscope, and the area corresponding to each microcolony was measured with ImageJ (Rasband, W.S., ImageJ, U. S. National Institutes of Health, Bethesda, Maryland, USA, https://imagej.nih.gov/ij/, 1997–2016).

Plate images were captured with the Canon EOS Rebel T3i camera (Canon, USA Inc.); the images were cropped and levels adjusted in Adobe Photoshop (Adobe Systems Incorporated, San Jose, CA). The levels of filipin fluorescence were uniformly adjusted using Zeiss AxioVision Rel 4.8 software. Unless otherwise stated, images were processed in Adobe Photoshop.

### Ergosterol analysis at a single cell level

Cells were harvested from liquid YPD, washed, and suspended in 1 ml PBS. Cells were then rotated in the dark with 10 μg/ml filipin III (from 5 mg/ml filipin stock in DMSO) (Cayman Chemical, Ann Arbor, MI) for 5 min. Cells were allowed to settle for 5 min in chamber slide prior to imaging. Importantly, the total time in filipin stain did not exceed 15 min. To ensure uniform filipin stain and to allow for comparison of treatments, wild type cells (H99) and cells expressing histone H4 tagged with the mCherry were incubated in conditions tested, and subsequently mixed together, and stained with filipin. Images were processed using ImageJ (https://imagej.nih.gov/ij/). To measure the intensity of filipin fluorescence, the plasma membrane marked by the filipin fluorescence was traced by a line 1 pixel thick, excluding an area where the daughter cell originates, and the intensity of each pixel was recorder along the traced line. Filipin value for each cell was calculated as an average of the pixel intensities from the entire traced line. To assess uniformity of the Filipin signal along the plasma membrane section, images were assessed by three independent evaluators (who had no knowledge which cell in an image was treated with FLC and which one was the control) and cells were classified as either patchy or having a smooth Filipin signal. All three evaluations were consistent indicating that classification of “patchy” was indeed based on a true difference between cells.

## Results

### Population of cells treated with fluconazole exhibits variable diminishment of ergosterol

**Figure 1:**
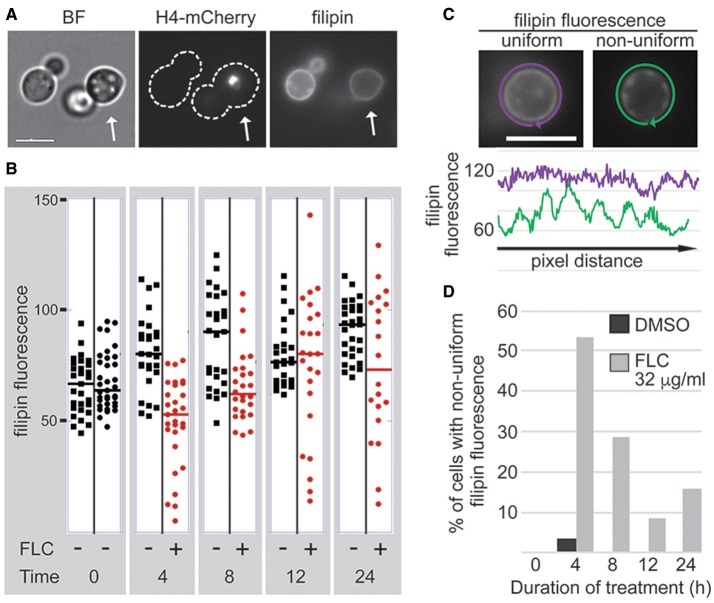
FLC treatment leads to unequal depletion of the plasma membrane ergosterol. The fluorescent dye filipin served as a proxy to examine the effect of FLC on the ergosterol content in the plasma membrane at a single cell level. **(A)** Strain expressing histone H4-mCherry was treated with 32 μg/ml FLC, whereas a strain lacking nuclear fluorescence was treated with DMSO and served as a control. The two strains were treated for indicated times. Prior to filipin staining strains were mixed in a 1:1 ratio, stained with filipin, and imaged immediately **(A)** Cells treated with FLC were recognized based on the H4-mCherry fluorescent signal (the cell on the right indicated by an arrow). **(B)** The average filipin fluorescence of cells treated with FLC exhibited increase in variability compared to DMSO-treated cells after 4 h of treatment. **(C,D)** FLC treatment leads to a non-uniform depletion of sterol from the plasma membrane within a single cell. **(C)** Filipin fluorescence was measured by tracing the fluorescent signal corresponding to the plasma membrane, and the intensity of the fluorescence of every pixel in the tracing path was plotted. Two representative cells are shown, DMSO-treated cell with the uniform fluorescence and the FLC-treated cell with the non-uniform fluorescence. **(D)** At least 19 cells from each sample were scored for the non-uniform filipin fluorescence. Bar in A and C corresponds to 5 μm.

The inhibitory effect of FLC has been associated with the depletion of ergosterol from the plasma membrane (Yoshida, 1988). Two non-exclusive possibilities may explain the heterogeneity of the response to FLC: (1) The degree of FLC-triggered depletion of ergosterol may vary from cell to cell or (2) The degree to which a cell is affected by a specific diminishment of ergosterol may vary from cell to cell. To test the first possibility, we utilized filipin as a proxy to estimate ergosterol levels in the plasma membrane of individual *C. neoformans* cells (Van Leeuwen et al., 2008). As filipin exhibits high sensitivity to light and oxygen, imaging the samples separately could potentially lead to false interpretations of the data. To resolve this issue, we employed two strains, a wild type (H99) strain and H99-derived strain that expressed histone H4 tagged with mCherry (Sutradhar et al., 2015). One of the strains was treated with FLC and the other served as a control. Prior to imaging, cells of the two samples were mixed, and filipin fluorescence was visualized for the mixed population. Scoring was executed taking into account mCherry fluorescent signal to differentiate between the samples (Figure 1A). For each evaluation, we conducted two reciprocal experiments to account for possible differences between the strains used (Figure 1B and Figure S1).

Untreated samples exhibited considerable variation in levels of filipin fluorescence between individual cells (Figure 1B, time 0). The variances for the two samples at time 0 were equal (at 0.05 significance level). A treatment with the inhibitory concentration of FLC (32 μg/ml) at 24°C caused an overall decrease of filipin fluorescence reaching an average fluorescence of ~56% of the control after 4 h (Figure 1B). Strikingly, after 4 h of FLC treatment the variance of filipin signal increased significantly as compared to the untreated control (443 vs. 91; these variances are unequal at 0.05 significance level), as some cells showed almost no detectable filipin fluorescence while others showed fluorescence levels close to those of the non-treated control (Figure 1B). We also noted that at 4 h of incubation over 50% of cells treated with FLC exhibited a non-uniform (patchy) fluorescence signal within the plasma membrane whereas less than 5% of control cells exhibited such irregular filipin fluorescence (Figures 1C,D). Further analysis was performed to investigate whether this non-uniform fluorescence signal was associated with a specific cell cycle stage and no correlation was found (data not shown).

Contrary to our expectation, treatment with FLC for 12 h or longer resulted in a less significant overall diminishment of the filipin fluorescence and also a smaller percentage of cells with non-uniform filipin fluorescence within the plasma membrane, as compared to the 4-h incubation (Figures 1B,D). In addition, differences in variance between the treated and the control sample were not significant for longer incubations except for 12 h where variance for treated sample was significantly higher (675 vs. 82). These data suggest that ergosterol levels vary from cell to cell in untreated population. Furthermore, FLC, at inhibitory concentrations, causes considerable diminishment of ergosterol from plasma membrane after 4 h of treatment and the effect is unequal between individual cells. Moreover, at the level of an individual cell, the depletion of ergosterol is non-uniform within the plasma membrane with areas exhibiting more or less diminishment of ergosterol. Together, these results point to considerable variation of levels to which FLC affects individual cells in the population with respect to the ergosterol content.

### A non-resistant subpopulation of cells forms colonies under inhibitory concentration of FLC without the fitness cost

Our analysis of filipin fluorescence in plasma membrane of individual cells suggested that a cell population exhibits variable ergosterol content before drug exposure and unequal diminishment of ergosterol during the initial exposure to FLC. We would predict that variability in sterol content should be reflected by heterogeneity with respect to the growth rate in the presence of FLC. A simple way to probe for the heterogeneity of cell population with respect to growth rate is to analyze the sizes of individual colonies on a semi-solid medium. In order to analyze the heterogeneity in response to the drug, we applied FLC at 24 μg/ml, which is a concentration below the heteroresistance level of 32 μg/ml established for the strain we utilized (Sionov et al., 2009).

**Figure 2:**
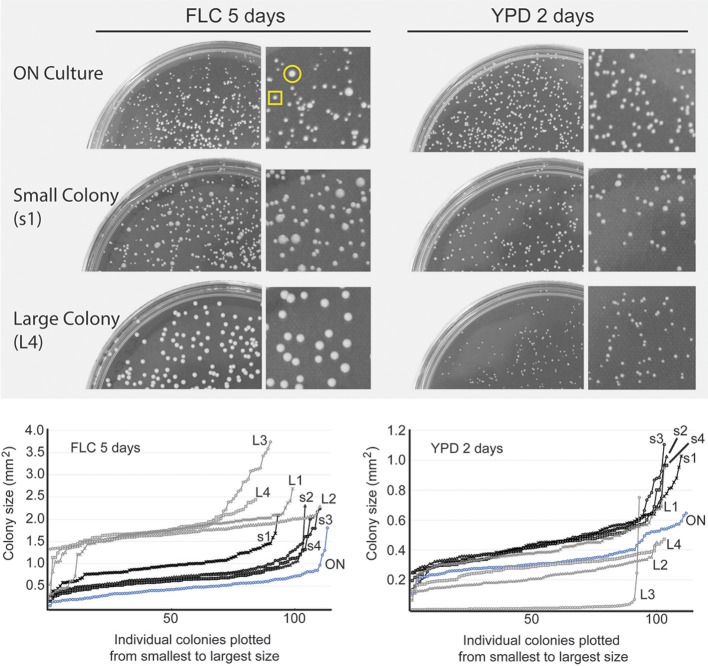
Heterogeneous response to FLC on a semi-solid media. Cells from the overnight culture (ON culture) were spread on YPD semi-solid media and YPD media containing 24 μg/ml FLC and incubated at 24°C. Cells incubated on FLC-containing media produced colonies that varied in size after 5 days, whereas cells incubated on YPD drug-free media formed colonies more uniform in size (top two plates). Four small colonies (representative indicated by yellow square) and four large colonies (indicated by yellow circle) were randomly picked from the FLC-containing plate and spread on YPD control media or YPD media containing 24 μg/ml FLC. Plates were photographed (representative images are shown) and sizes of the resulting colonies from areas containing ~ 100 colonies were analyzed and plotted. Next to each plate, a magnified area is shown. Cells obtained from small colonies (black lines, s1-4) displayed similar colony size heterogeneity on FLC media compared to the initial population (ON) (left graph) and largely retained their ability to proliferate when grown on YPD (right graph). Cells derived from big colonies (gray lines, L1-4) displayed smaller colonies on YPD and larger, more homogenous colonies on FLC-containing media.

An overnight culture plated on drug-free YPD rich semi-solid medium grew into colonies relatively uniform in size after 2-day incubation at 24°C (Figure 2, ON culture). An exposure of the same overnight culture to FLC at 24 μg/ml resulted in more variable colony sizes (Figure 2). After 5 days of incubation at 24°C, the effect of FLC ranged from a significant inhibition of proliferation (single cells or micro-colonies not visible by naked eye; data not shown) to growth retardation that resulted in visible colonies of various sizes (Figure 2).

Did all colonies that were visible after 5 days on FLC-supplemented media contain cells that were resistant to FLC? To address this question, we picked at random 4 relatively small, and 4 relatively large colonies and re-plated the cells on media containing 24 μg/ml of FLC at 24°C. In addition, the same samples were also plated on YPD drug-free media to test for potential growth retardation that would be expected if the cells were aneuploids (Sionov et al., 2010). Cells from all 4 small colonies, when re-plated on FLC-containing media resulted in colonies of various sizes and single cells after 5 days, indicating a range of inhibition characteristic of a population that is not resistant to the drug, although the visible colonies were relatively larger as compared to the initial culture plated on FLC media (Figure 2 and data not shown). In contrast, cells from the large colonies grew significantly better on new FLC plates (Figure 2). Interestingly, when the distribution of colony sizes was assessed for the cells re-plated on the control YPD media, cells from three out of four large colonies grew relatively slower as compared to cells obtained from small colonies (Figure 2). Notably, cells from one large colony (indicated as L3 in Figure 2 graph) exhibited a fraction of colonies that were particularly large on FLC-supplemented media, while the majority of cells derived from this colony formed significantly smaller colonies on YPD drug-free media. Thus, for the cells derived from FLC-supplemented media, their ability to proliferate subsequently on new FLC-containing media correlates inversely with their proliferation rate on drug-free media. These results suggest that when the original overnight culture was spread on FLC-containing media only a fraction of colonies, likely the largest colonies, contained cells resistant to FLC used at concentration equal to 24 μg/ml, potentially through becoming aneuploids (as judged indirectly based on relatively slower growth on drug-free media). The remaining, smaller colonies represented cells that were not resistant to the drug yet managed to grow into visible colonies without a loss of fitness. We designate these non-resistant cells as survivors in contrast to cells that did not proliferate and truly resistant cells that formed relatively large colonies. Our data suggest that an asynchronous population of *C. neoformans* exhibits an intrinsic heterogeneity that is reflected by variable growth rate in the presence of FLC. Furthermore, upon exposure to subinhibitory concentration of FLC a fraction of the cell population develops into colonies that are not resistant to the drug yet manage to grow at a rate that is slower than resistant cells.

### *C. neoformans* cells display a normal distribution with respect to colony size on semi-solid, drug-free media

**Figure 3:**
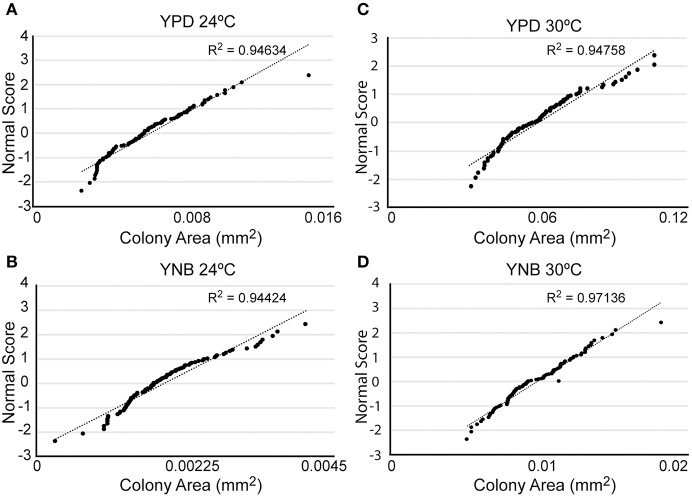
Cells not exposed to FLC exhibit a normal distribution of colony sizes. Cells were plated on YPD semi-solid media **(A,C)** or YNB semi-solid media **(B,D)** and incubated at either 24°C **(A,B)** or 30°C **(C,D)**. The size of ~100 colonies was measured and plotted against a normal distribution of numbers (correlation coefficient R^2^ indicates correlation between distribution of colony sizes and the normal distribution). Cells grown on YPD exhibited relatively larger colonies as compared to cells grown on YNB, and 30°C led to relatively larger colonies as compared to 24°C.

What could account for the heterogeneity of response to FLC? Given that the variable response can be detected already after 4 h of incubation with FLC (Figure 1), this heterogeneity is likely an intrinsic feature that characterizes the cell population prior to the drug exposure. Potentially such an intrinsic heterogeneity may be reflected by variable growth rates of individual cells even under unperturbed growth conditions. To examine this possibility, cells were spread on semi-solid rich YPD drug-free media, and the size distribution among randomly sampled ~100 micro-colonies detected under the microscope after 24 h of incubation at 24°C was analyzed (Figure 3A). This analysis revealed that under drug-free conditions individual colonies of *C. neoformans* exhibit distribution of sizes that fits significantly to a normal distribution (*R*^2^ = 0.95). Accordingly, the population of cells contained two small subpopulations characterized by opposite extreme behaviors with respect to growth rate; a small fraction grew significantly slower and a second small fraction grew significantly faster as compared to the majority of cells in the population. Such a distribution of growth rates among the cells may reflect how the cell population would respond to FLC treatment.

### Rate of growth influences the response to FLC

We hypothesized that the size of the colony that a given cell develops into in the presence of FLC correlates inversely with the rate of growth of a colony that would develop from this cell in drug-free conditions. For example, a fraction of cells that are most successful in proliferating during the initial exposure to the inhibitory concentration of FLC would be cells that are predisposed to exhibit relatively slower growth in drug-free conditions. If that were the case, we would predict that introducing conditions that normally (without the drug) promote slower growth should lead to an overall improved survival in the presence of FLC.

**Figure 4:**
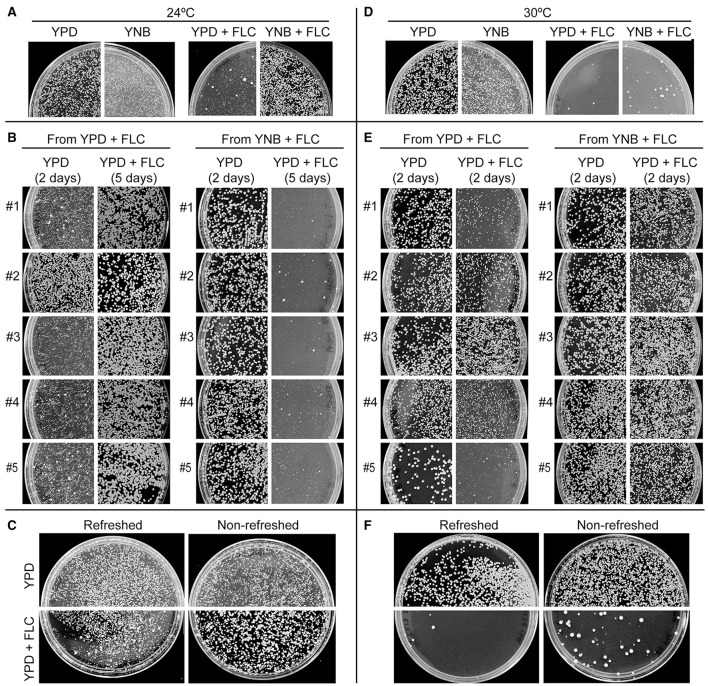
The response of FLC depends on temperature, media composition, and nutrient availability. **(A,D)** Incubation of cells on minimal medium YNB supplemented with 32 μg/ml FLC led to a significantly larger number of colonies at both 24°C and 30°C as compared to cells incubated on rich YPD media containing 32 μg/ml FLC. Incubation at 30°C led to an increased FLC susceptibility on both YPD and YNB media as compared to 24°C. **(B,E)** Five relatively big colonies obtained from either YPD or YNB FLC-containing media (shown in **A**,**D**) were re-plated on YPD semi-solid media with and without 32 μg/ml FLC. **(C,F)** Cells that were grown for 7 days in YPD liquid media without refreshing the media exhibited more colonies when subsequently spread on semi-solid media containing 32 μg/ml FLC as compared to cells that were at the exponential phase of growth prior to FLC exposure. This effect was significant at both 24 and 30°C.

Comparison of colony sizes indicated that cells incubated on drug-free semi-solid defined minimal media (YNB) proliferate significantly slower as compared to those cultured on rich YPD media (Figures 3A,B). Consistent with our hypothesis, plating cells on YNB minimal medium supplemented with 32 μg/ml of FLC (YNB+FLC) led to a significant increase in the number and size of colonies as compared to the YPD supplemented with the same amount of FLC (YPD+FLC) (Figure 4A). Randomly picked, relatively large colonies grown at 24°C on YPD+FLC media contained mostly resistant cells, as majority of these cells formed robust colonies when re-plated and incubated under the same conditions (Figure 4B). In contrast colonies randomly obtained from YNB+FLC media incubated at 24°C were not resistant to subsequent exposure to FLC in analogous conditions (Figure 4B). This suggests that the majority of colonies derived from the YNB+FLC plates are not resistant to FLC. Consistent with these findings, 4 out of 5 randomly picked large colonies from the YPD+FLC plates grew relatively slower when re-plated on the YPD drug-free media, whereas colonies obtained from the YNB+FLC plates contained cells that grew relatively better when spread on the YPD media (Figure 4B). These findings suggest that conditions that promote slower growth lead to an increase in the number of colonies that contain non-resistant cells (survivors).

Another condition that results in an overall lower proliferation rate is nutrient deprivation and increased cell density when the culture approaches stationary phase of growth. We tested whether incubation of cells without refreshing the medium would influence subsequent growth on media containing FLC. Strikingly, prolonged incubation of cells in liquid rich YPD medium before plating on FLC-containing media led to an overall increase in colony number and size as compared to cells that were grown exponentially prior to FLC exposure (Figure 4C). The effect was already observed for cells incubated for 2 days (data not shown) in liquid YPD prior to FLC treatment and was more pronounced for longer incubation times (Figure 4C). This result suggests that in contrast to cells at the exponential phase of growth, cells derived from a nearly stationary phase culture are relatively less sensitive to FLC and more likely to develop into colonies.

Together our data suggest that external conditions that lead to an overall slower growth result in a better survival in the presence of FLC. We would predict that generating external conditions, which lead to an increased growth rate should elicit an opposite effect. One such condition is elevated temperature, which in case of *C. neoformans* results in increased proliferation rate (Figure 3). The intrinsic level of heteroresistance for the *C. neoformans* var. *grubii* (serotype A) strain H99 has been estimated at 32 μg/ml FLC at 30°C on semi-solid YPD media (Sionov et al., 2009). We confirmed that when H99 cells were incubated with FLC under these conditions, only a very small fraction developed into visible colonies (up to ~15 colonies were visible without magnification after 5 days on a plate on which ~10,000 cells were plated), in contrast to cells incubated at 24°C (Figure 4D compared to Figure 4A). Consistent with previous reports, all of the visible colonies were resistant to the drug (most cells derived from a given colony grew upon subsequent plating on the same FLC concentration) and were likely aneuploids (most cells derived from a given colony grew slower on YPD drug-free media as compared to control cells, which were never exposed to the drug) (Figure 4E) (data not shown). These findings suggest that higher temperature leads to more susceptibility to FLC. Interestingly, cells derived from all randomly picked colonies that developed at 30°C on YNB 32 μg/ml FLC media were resistant to the drug (Figure 4E). Notably, while the effect of YNB at 30°C was significant (more colonies as compared to YPD 32 μg/ml at 30°C), the number of visible colonies was significantly smaller as compared to YNB 32 μg/ml FLC plates incubated at 24°C. This result suggests that the higher temperature resulted in an overall lower percentage and a decrease in colony size of “survivors,” while maintaining (or potentially increasing) the number of resistant colonies. Consistent with these findings, higher temperature incubation also led to a decrease in colony numbers of cells derived from nearly stationary culture (Figure 4F).

We also tested the effects of temperature and media type on heterogeneity of the response to FLC of another serotype A strain, KN99a (mating type **a**), which has been derived from the H99 (mating type α) through 10 genetic backcrosses based on unrelated serotype A strain isolate 125.91 (Nielsen et al., 2003). While KN99a is not genetically identical to H99 it is nonetheless significantly congenic and can serve to test the effect of the mating type on the FLC sensitivity and the influence of the environment. We noted that KN99a was more sensitive to FLC as compared to H99 as incubation on semi-solid media supplemented with 32 μg/ml FLC resulted in no colonies even at 24°C. However, exposure of KN99a to 16 μg/ml FLC at 24°C revealed heterogeneity in colony sizes similar to strain H99 incubated with 32 μg/ml FLC at 24°C (data not shown). Furthermore, 2 relatively large colonies were resistant to subsequent exposure to 16 μg/ml FLC at 24°C and 2 relatively small colonies were not (data not shown). Similarly to H99, incubation of KN99a at 30°C on semi-solid media supplemented with 16 μg/ml FLC lead to a significant decrease in colony number and only relatively large colonies were observed that were resistant to FLC upon subsequent plating. Furthermore, incubation of KN99a on YNB media supplemented with 32 μg/ml FLC resulted in significantly improved growth at both 24 and 30°C (data not shown). Thus, the effects of the temperature and the media type on proliferation in the presence of FLC were common to both mating types of the serotype A type.

While 30°C has been established as a standard growth condition for drug susceptibility testing of *C. neoformans*, the temperature relevant to infection is at least 37°C (Archibald et al., 2004). Therefore, we tested the effect of 37°C on colony growth in the presence of FLC. When a culture that was maintained initially in drug-free liquid YPD media at 24°C was subsequently incubated on drug-containing YPD semi-solid media at 37°C, a complete inhibition of growth was observed at a FLC concentrations as low as 1 μg/ml with no occurrence of resistance to the drug (out of 10,000 cells plated, no colonies were observed after 5 days at 37°C). Such a significant change in sensitivity to FLC (from 32 to 1 μg/ml) could stem from a synergistic effect between FLC and a significant and sudden change in temperature (from 24 to 37°C) and media type (liquid to semi-solid). Indeed, when cells were initially grown for 24 h at 37°C in liquid YPD drug-free media and subsequently incubated at 37°C on semi-solid media containing FLC, the concentration of 1 μg/ml was no longer inhibitory (data not shown). However, under those conditions the level of heteroresistance was estimated at ~5 μg/ml FLC, which is significantly lower than 32 μg/ml established at 30°C (data not shown).

A previous study has suggested that already after ~10 h of exposure, FLC leads to an increase in ploidy in a significant population of *C. neoformans* (Altamirano et al., 2017). Notably, we found that for a given concentration of FLC, the effect on ploidy was more significant with an increase in temperature (Figure S2). Together, these findings suggest that temperature is a critical factor that modulates the response of *C. neoformans* to FLC and influences the level of heteroresistance. These data are consistent with the important role of growth rate in the susceptibility to FLC.

### Age of cells may be one of the intrinsic factors contributing to heterogeneity of the response to FLC

**Figure 5:**
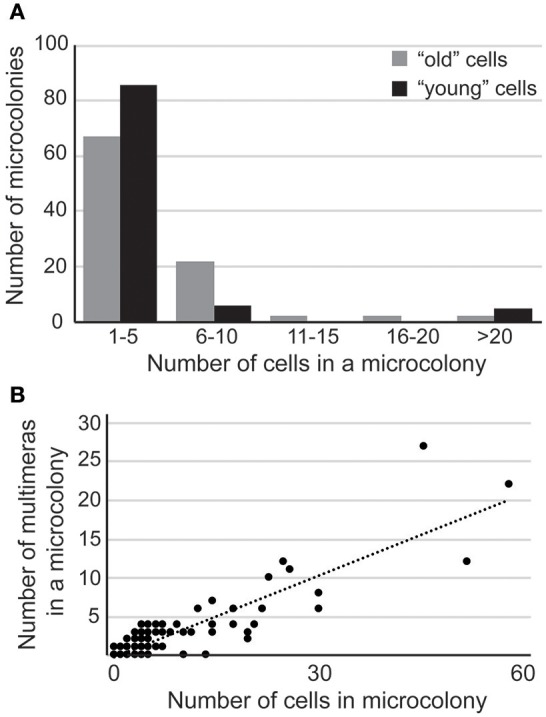
**(A) “**Young” cells display more sensitivity toward FLC. Pre-selected unbudded cells were biotinylated and released into YPD for 4 h. Cells were then stained with streptavidin-conjugated FITC and sorted based on FITC-positive (older cells) or FITC-negative (young cells) signal. Young and older cells were spread on YPD media supplemented with 32 μg/ml FLC, incubated at 30°C, and after 6 days 100 colonies were dissected and number of cells in each colony was counted to assess degree of proliferation in the presence of FLC. The majority of young cells display an overall higher sensitivity to FLC as compared to the mixed population. **(B)** Multimera morphology is not sufficient, but may be essential for the development of resistance to FLC. Cells were incubated on YPD media containing 32 μg/ml FLC at 30°C for 6 days. One hundred random colonies/cells were dissected and the number of normal and multimeric cells was counted in each micro-colony.

A culture of *C. neoformans* contains cells with various life spans. Therefore, age may potentially be one of the factors contributing to the heterogeneity in response to FLC. To test this possibility, we labeled cell walls with the fluorescent probe, Fluorescein Isothiocyanate (FITC), washed away the staining reagent, and incubated the culture for 3.5 h in drug-free media such that the newly grown and detached daughters were no longer labeled. Next, cells that were FITC-positive (a mix of cells with variable age) and FITC-negative (more homogenous with respect to age, relatively young cells) were separated using fluorescence-assisted cell sorter (FACS). Subsequently, the separated fractions were plated on 32 μg/ml FLC YPD semi-solid media and incubated at 30°C. Strikingly, in contrast to the mixed population, the “young” cells exhibited less heterogeneous response and were overall more sensitive to FLC as judged based on the distribution of colony sizes (Figure 5A). This finding suggests that the age of cells in the population is one of the factors contributing to the heterogeneous response to FLC.

### Multimera morphology is contributing positively to the survival in the presence of FLC

According to previous studies, *C. neoformans* cells that develop resistance to the initial inhibitory concentration of FLC are aneuploids typically with disomic chromosomes 1, and/or 4 (Sionov et al., 2010). Studies in *Candida albicans* suggested that a prerequisite to aneuploidy-derived resistance to FLC is the formation of aberrant multimeric cells that give rise to tetraploid populations, which subsequently undergo chromosomal loss (Harrison et al., 2014). A recent study also demonstrates that FLC leads to cell separation defect followed by development of a second daughter cell, which results in formation of multimeric cells in *C. neoformans* (Altamirano et al., 2017). Altamirano et al. reported that cells with increased ploidy and aberrant morphology exhibit overall improved growth in the presence of FLC (Altamirano et al., 2017). If the ability of *C. neoformans* to grow in the presence of FLC were dependent on the formation of multimeric cells, we would expect a correlation between the colony size and the presence of multimeric cells within a colony.

In order to assess the response of individual cells to FLC, we plated cells on rich YPD media containing 32 μg/ml FLC, incubated at 30°C for ~6 days, and evaluated growth under the microscope. Out of 10,000 cells that were plated, 12 colonies were visible consistent with previously established heteroresistance level (Sionov et al., 2009). Microscopic observation of the remaining cells present on the medium revealed single cells as well as micro-colonies of various sizes. To gain more insight into the morphology of the cells we scored or dissected 100 random cells or colonies, respectively, and analyzed cellular morphology of all the cells from each micro-colony (Figure 5B). Among 100 scored, we found four single cells suggesting that approximately 96% of cells that were originally plated have divided at least once within 6 days of incubation. These single cells exhibited normal morphology (were not multimeras). In contrast, microcolonies contained variable number of multimeric cells, which correlated with the colony size (Figure 5B; *R*^2^ = 0.77). Thus, our data suggest that formation of multimeric cells may promote proliferation and reflect the heterogeneity of the response to FLC.

While the aneuploid state associated with the resistance is not stable, FLC-resistant aneuploid cells should persist under the selective pressure of the drug (Sionov et al., 2010). This may potentially indicate that the cells at the edge of the visible resistant colony grown under selective pressure of FLC should exhibit wild type morphology. However, the analysis of the edge of resistant colonies (found on YPD media containing 32 μg/ml FLC and incubated at 30°C for ~6 days) contradicted this possibility as it showed the presence of multimeric cells (data not shown). This finding was further supported by the flow cytometry analysis of sizes and the complexity of morphology of the cells derived from YPD plates supplemented with 32 μg/ml FLC and incubated at 30°C. The results of this analysis suggest that both small (survivors) and large (resistant) colonies contain significant fraction of enlarged and morphologically changed cells (Figure S3). Thus, colonies consisting of resistant cells are morphologically heterogeneous suggesting that growth of the resistant colony involves a continual evolution of resistance based on formation of multimeric cells. While multimeric cells may not be sufficient for generation of FLC resistance, their formation may positively influence the survival of initially formed microcolonies in the presence of FLC.

**Figure 6:**
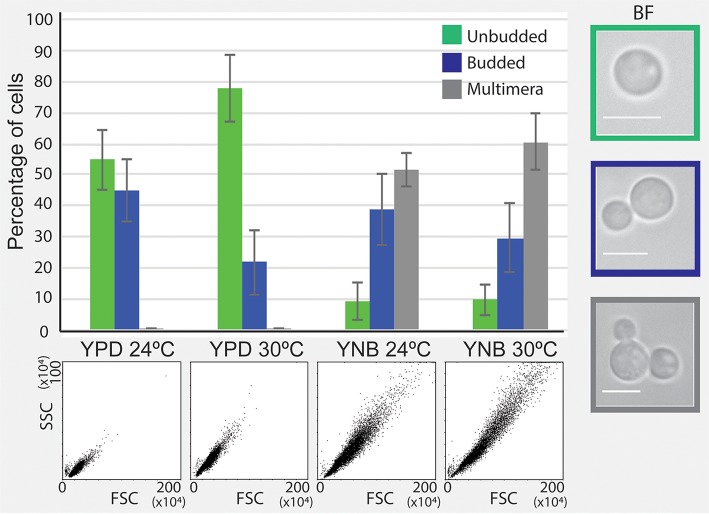
Incubation in minimal YNB media results in the formation of multimeric cells. Cells were incubated in YPD and YNB media at 24 and 30°C and cellular morphology was assessed by flow cytometry and brightfield (BF) microscopy. A significant fraction of cells grown in YNB media exhibited multimera morphology, in contrast to cells incubated in YPD.

If multimeric cells were indeed contributing to a better survival in the presence of FLC, we would predict that conditions that lead to a delay in daughter cell separation (a phenomenon that leads to multimeric state) may promote improved proliferation in the presence of FLC. Interestingly, a culture grown in YNB medium without FLC consisted of a significant number of multimeras at both 24 and 30°C (Figure 6). Therefore, the increased survival on YNB semi-solid media may be due to increased multimera formation and may be common to other conditions that promote slow proliferation and improve growth in the presence of FLC. These results suggest that while conditions that promote slower proliferation generally improve survival in the presence of FLC, the mechanisms through which this occurs may vary depending on a specific condition.

## Discussion

Our data and previous studies suggest that *C. neoformans* genetically homogenous population consists of cells that differ in their potential to proliferate when first exposed to FLC (Sionov et al., 2010). Since we observed similar responses for two serotype A strains, H99 (mating type **α**) and KN99a (mating type **a**), our results suggest that phenotypic heterogeneity with respect to proliferation in the presence of FLC and the effects of the environment are common to other serotype A (*C. neoformans* var. *grubii*) strains and do not depend on the mating type. However, given a high level of genetic similarity between H99 and KN99a, it remains possible that other, unrelated serotype A strains may behave differently in the presence of FLC. On the other hand, serotype D strain JEC20 (*C. neoformans* var. *neoformans*), although it was relatively more sensitive to FLC, also exhibited heterogeneity in colony sizes upon exposure to 4 μg/ml FLC (data not shown). Other studies suggest that the effect of temperature on proliferation in the presence of FLC similar to the effect described here is common to various unrelated setotype A strains (Mondon et al., 1999; Pettit et al., 2010). Furthermore, distantly related species *Cryptococcus gatii* also exhibits heterogeneity with respect to growth rate in the presence of inhibitory concentration of FLC (Varma and Kwon-Chung, 2010). Subsequent investigations should establish if the influence of various factors on heterogeneity of the response to FLC are common to *C. neoformans* var. *grubii* and other *Cryptococcus* species.

We propose that the potential of cells to proliferate in the presence of FLC depends, at least in part, on the ability to retain ergosterol at the plasma membrane. While we have not tested whether this relationship is causal, previous studies have shown that the inhibitory effects of FLC on cellular proliferation are attributed to ergosterol depletion (Yoshida, 1988). Most studies have assessed the effects of FLC on ergosterol levels after an overnight treatment with FLC. Moreover, the actual dynamics of the depletion of ergosterol has not been well-established in any model organism. Furthermore, most studies assessed the total amount of ergosterol extracted from an entire cell population. The only study that describes ergosterol content at the level of an individual cell assessed ergosterol levels based on the fluorescence of the sterol-binding agent filipin in *Penicillium discolor* and *Saccharomyces cerevisiae* (Van Leeuwen et al., 2008). Filipin staining utilized here as a proxy to assess the ergosterol content suggests an overall diminishment of plasma membrane ergosterol in FLC-treated populations that is non-uniform between individual cells. Interestingly, diminishment appeared also non-uniform within a single cell. Specifically, patches of higher fluorescence were distributed throughout the plasma membrane. This finding suggests that specific areas within the plasma membrane are more resistant to diminishment of ergosterol. One such area could be the sterol-rich lipid raft domains described previously in other cell types (Siafakas et al., 2006; Alvarez et al., 2007).

At 12 and 24 h of incubation with FLC the overall diminishment of filipin fluorescence was less significant as compared to 4-h time-point. One potential explanation could be that longer incubations may lead to a compensatory synthesis and incorporation of alternative sterols into the plasma membrane and an overall change in sterol composition (Ghannoum et al., 1994). An alternative and non-exclusive possibility is that longer incubations lead to re-establishment of ergosterol in cells that have gained the resistance potential to continue proliferating in the presence of the drug.

The degree to which FLC affects the levels of ergosterol may depend on multiple factors that contribute to the phenotypic heterogeneity within the population. Phenotypic heterogeneity and its role in FLC heteroresistance has not been addressed in *C. neoformans*. In clonal single-celled organisms, phenotypic heterogeneity is largely attributed to gene expression “noise” or differential gene expression, characteristic for populations of cells that differ in cell cycle progression and/or cellular aging (Sumner et al., 2003; Cohen et al., 2013). In addition to intrinsic sources of phenotypic heterogeneity, such as ergosterol levels, extrinsic factors such as nutrient availability, population density, and oxidative stress have all been shown to promote persistence and likely play a role in FLC heteroresistance (Holland et al., 2014). For instance, our other studies suggest that FLC treatment leads to an increase in Reactive Oxygen Species (ROS), which contribute to the inhibition of growth (submitted). Upon FLC exposure, individual cells within the population may vary in the levels of ROS. Similar to the retention of ergosterol, levels of ROS may also depend on multiple factors. In addition, we cannot exclude the possibility that the levels of ergosterol and ROS are interdependent.

Based on our data and previous findings, we propose that each cell in the population is characterized by a net potential (derived from multiple factors) to proliferate in the presence of FLC. These potentials are specifically distributed among cells within the population. We postulate that the shape of this distribution depends on intrinsic as well as extrinsic factors. Critical extrinsic factors that were characterized here include drug concentration, temperature, and the availability of nutrients. Our data suggest that an intrinsic factor that influences the response to FLC is replicative age of the cell. Specifically, a population consisting of mostly young cells exhibited a response that displayed relatively lower heterogeneity and an overall higher sensitivity to FLC. Interestingly, such a population still contains a small fraction of cells capable of becoming resistant to FLC. Consistent with our findings, it has been reported that *C. neoformans* cells that are old with respect to the replicative life span are more resistant to FLC (Bouklas et al., 2013).

**Figure 7:**
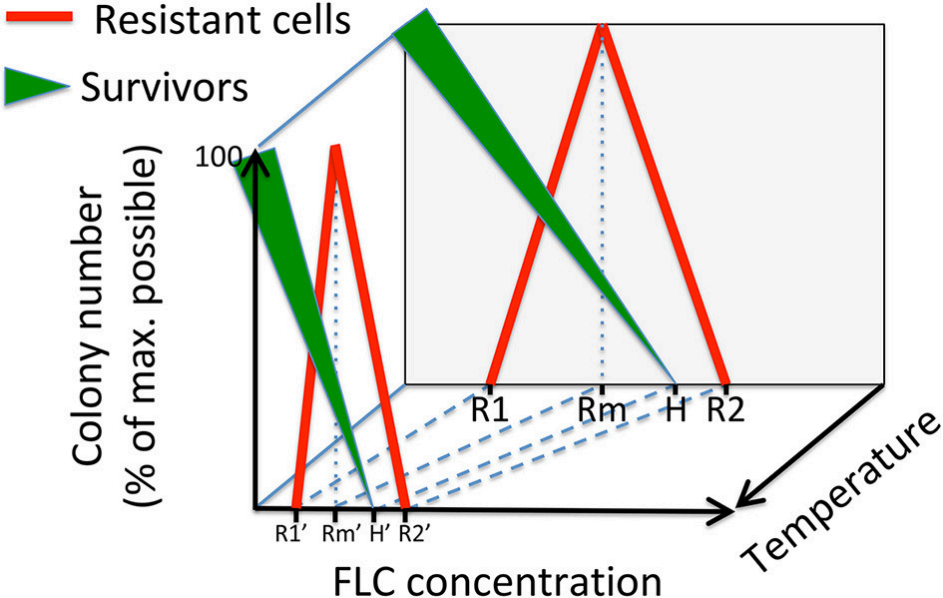
A model depicting the effects of the FLC concentration and the temperature on the distribution of two types of colonies of *C. neoformans* that are visible on semi-solid media. Green triangular lines illustrate colonies consisting of survivors, which are cells not resistant to FLC. The thickness of the green line refers to the average colony size. The red line illustrates colonies consisting of mostly resistant cells. Specific concentrations of FLC included in the model are: R1, minimum concentration at which resistant cells develop; Rm, concentration at which maximum resistant colonies develop; H, heteroresistance level (lowest concentration at which no survivors form visible colonies); R2, lowest concentration at which no colonies are observed.

As schematically depicted in Figure 7, at a given temperature, a population of cells that is treated with FLC is divided into those that do not grow into visible (by naked eye) colonies after ~5 days (significantly inhibited), those that form visible colonies consisting of non-resistant cells (survivors, depicted as green triangular line), and those that form colonies composed of mostly resistant cells (red line). As the concentration of FLC increases, the number and size of colonies consisting of survivors decrease until no visible colonies are observed even after ~5 days (FLC heteroresistance level, depicted as H). We postulate that colonies consisting of resistant cells appear only when concentration of FLC is at a necessary minimum (depicted as R1). The number of resistant colonies reaches maximum at the FLC concentration depicted as Rm. Based on our data, we propose that at concentration Rm, colonies of survivors are visible albeit their size is relatively smaller as compared to lower concentrations. According to this model, a minimum concentration of FLC at which survivors are no longer observed and resistant colonies are still present (depicted as H) is the heteroresistance level as described previously (Sionov et al., 2009). Finally, at a concentration of FLC depicted as R2 no colonies are visible with a naked eye even after 5 days of incubation.

While, according to this model, the size of colonies consisting of survivors decreases with increasing FLC concentrations (depicted as the width of the green triangular line), the size of colonies consisting of resistant cells is relatively uniform (red line), consistent with the definition of the resistant state. While our model depicts linear relationships between colony numbers (or colony size for the survivors) and concentration of FLC, in reality these relationships are likely non-linear. The exact function describing these relationships will depend on the nature of multiple factors (intrinsic and extrinsic) influencing cell growth in the presence of FLC.

Our findings suggest that temperature is one of the critical extrinsic factors impacting the distribution of the potential to proliferate in the presence of FLC. Temperature has been shown to influence transcriptional responses in *C. neoformans* and its effects are likely pleiotropic (Kraus et al., 2004). Previous findings suggested that the susceptibility of *C. neoformans* to FLC increases with the elevation of temperature. Pettit et al. noted that biofilms formed by *C. neoformans* were more susceptible to FLC and the MIC's of the planktonic yeast cultures were lower at 35°C as compared to 30°C (Pettit et al., 2010). Mondon et al. demonstrated that a clinical isolate of *C. neoformans* fully resistant to 64 μg/ml FLC at 30°C forms a reduced number of colonies at 35°C, and no colonies at 37 or 40°C (Mondon et al., 1999). Our data suggest that the concentrations depicted in our model as R1, Rm, H, and R2 shift toward lower values as temperature of incubation increases (Figure 7). We propose that other factors, will shift these concentration values in either of the two directions depending on the specific factor. Thus, these concentration values for a given population of cells under specific conditions would result from the net interaction between all factors.

One factor that is influenced by various conditions (extrinsic and intrinsic) is rate of proliferation. Three extrinsic factors we analyzed in this study are temperature, the type of growth media (rich medium versus minimal medium) and the phase of growth (exponential versus stationary), all of which influence the proliferation rate. Specifically, lower temperatures, higher cell densities (cells at or approaching stationary phase) and growth in minimal media (as opposed to rich media) result in an increase in the number and size of colonies consisting of survivors and resistant cells. Thus, for all three extrinsic factors studied here, the conditions that result in shift of concentrations R1, Rm, H, and R2 toward higher values are all associated with slower growth rate in drug-free media, suggesting that conditions which result in slower growth/metabolism promote survival in the presence of FLC. Elucidation of mechanisms underlying increased survival of slow growers during FLC treatment requires further investigations. One potential explanation for the increased survival of slow-growers may be decreased ATP production and mitochondrial function in slow-growing cells, as has been observed during persistence in bacterial populations (Shan et al., 2017). Consistent with this possibility, a study in *C. albicans* showed that ATP levels serve as a good predictor for FLC susceptibility (Kretschmar et al., 1996). Populations of cells characterized by higher ATP levels exhibited lower minimum inhibitory concentration for FLC (Kretschmar et al., 1996). Additionally, a study in *C. neoformans* showed that cells treated with tetracycline, a mitochondrial translation inhibitor, were more resistant to FLC (Panepinto et al., 2010). The correlation between ATP levels of individual cells and their survival in FLC should be further studied as targeting cellular metabolism during FLC treatment could improve current therapeutic approaches.

Microbial pathogens may become less susceptible to an inhibition imposed by a drug through reducing the rate of the process that is the primary drug target (Shan et al., 2017). Such cells are not resistant to the drug, but they possess a better chance of survival during the initial exposure to the drug and therefore exhibit a higher chance to develop into drug-resistant populations. While the effects of FLC are likely pleiotropic, a prominent result of FLC treatment in *C. neoformans* is inhibition of budding (Altamirano et al., 2017). It is therefore likely that conditions that promote slower growth result in less susceptibility to FLC. Alanio et al. showed that a subpopulation exhibiting slower growth rate, thought to be dormant cells, exist *in vitro* and *in vivo* in *C. neoformans* populations (Alanio et al., 2015). These dormant cells could potentially promote survival during FLC treatment.

Our data based on microscopic examination suggest that at concentrations of FLC depicted in our model as between R1 and R2, a large fraction of cells (increasing as the concentration of FLC approaches R2) form micro-colonies (not visible by naked eye after ~5 days). These micro-colonies typically consist of multimeric cells, but the presence of multimeric cells does not ensure resistance. This however does not exclude the possibility that the formation of multimeras is necessary for the subsequent development of resistance. For instance, multimeric cells may promote sufficient proliferation to increase the number of stochastic events of aneuploidy formation out of which only a small percentage provides sufficient growth advantage and resistance to the particular concentration of the drug. Moreover, multimeras may be necessary for the maintenance of the resistant state. This is supported by the observation that multimeras are present at edges of the resistant colonies. This finding also suggests that a colony consisting of resistant cells is heterogeneous and contains cells at various resistance levels. It is possible that as the resistant colony grows, a continual process of gaining and losing the resistance continues within the colony, and a distribution of cells with various levels of resistance characterizes the colony. Significant presence of multimeric cells in drug-free YNB media further supports positive role of multimeras in survival in the presence of FLC. However, we cannot exclude the possibility that multimera formation could be a side effect of slow growth rather than a factor contributing to better survival. For instance, cells derived from nearly stationary culture are not multimeras (data not shown). Additionally, colonies grown on YNB semi-solid media at 30°C appeared to grow at approximately the same rate as cells grown on YPD semi-solid media at 24°C, which did not promote multimera formation (Figures 3A,D and 6).

Recent findings suggest that in cancer cells exposed to an anticancer drug an epigenetic reprogramming takes place rendering a subpopulation of cells less sensitive to the drug (Shaffer et al., 2017). While our study suggest that a preexisting heterogeneity within the population of *C. neoformans* determines the response of individual cells to the drug, it is possible that in a population under specific growth conditions and drug concentration, a subset of cells may possess the ability to divide and/or reprogram epigenetically, so that these cells lead to progeny able to acquire the resistant state subsequently.

Grossman and Casadevall highlighted that *C. neoformans* undergoes unique changes during infection compared to laboratory *in vitro* conditions, making *in vitro* analyses suboptimal in predicting outcomes in infection models (Grossman and Casadevall, 2017). Our study highlights another barrier to effective therapeutic strategies based on standardized *in vitro* susceptibility testing. The variability of the response to FLC due to extrinsic and intrinsic factors reported here should help to better understand phenotypic heterogeneity in *C. neoformans* and its role during FLC treatment.

## Author contributions

SA and LK: conception and design of the experiments. Experiments were performed by SA, except for experiments described in Figure 2 that were performed by CS. SA and LK: evaluation and analysis of the results and manuscript writing, and final approval of manuscript.

### Conflict of interest statement

The authors declare that the research was conducted in the absence of any commercial or financial relationships that could be construed as a potential conflict of interest.

## Supplementary material

Figure S1 FLC treatment leads to unequal depletion of the plasma membrane ergosterol. The fluorescent dye filipin served as a proxy to examine the effect of FLC on the ergosterol content in the plasma membrane at a single cell level. A strain H99 was treated with 32 μg/ml FLC, whereas a strain expressing histone H4-mCherry was treated with DMSO and served as a control. This represents a reciprocal of the similar experiment depicted in Figure 1. The two strains were treated for indicated times. Prior to filipin staining strains were mixed in a 1:1 ratio, stained with filipin, and imaged immediately. The average filipin fluorescence of cells treated with FLC exhibited increase in variability compared to DMSO treated cells after 4 h of treatment.

Figure S2 Increasing the incubation temperature leads to a more pronounced ploidy increase in FLC-treated cells. Cells were treated with DMSO (control) or FLC at indicated concentrations (μg/ml) for indicated times at either 24 (RT) or 30°C. Samples were fixed, stained with propidium iodide and analyzed by flow cytometry.

Figure S3 Analysis of the cell size and morphological complexity of cells. Cells were grown on YPD drug-free plates for 2 days or YPD plates supplemented with 32 μg/ml FLC for 5 days at 30°C. Cells from individual colonies were analyzed by flow cytometry to assess their sizes and morphological complexity. Cells obtained from the control YPD plate (ON culture) are uniform and relatively small. In contrast, cells obtained from the small colony (survivors) and the large colony (resistant cells) from the FLC-supplemented media show significant shift toward larger size and complexity.
