# Supplementary figures and images for: Colony and Single Cell Level Analysis of the Heterogeneous Response of Cryptococcus neoformans to Fluconazole
Source: Front Cell Infect Microbiol. 2018 Jun 19;8:203. doi: 10.3389/fcimb.2018.00203 (PMC6018158; doi:10.3389/fcimb.2018.00203)

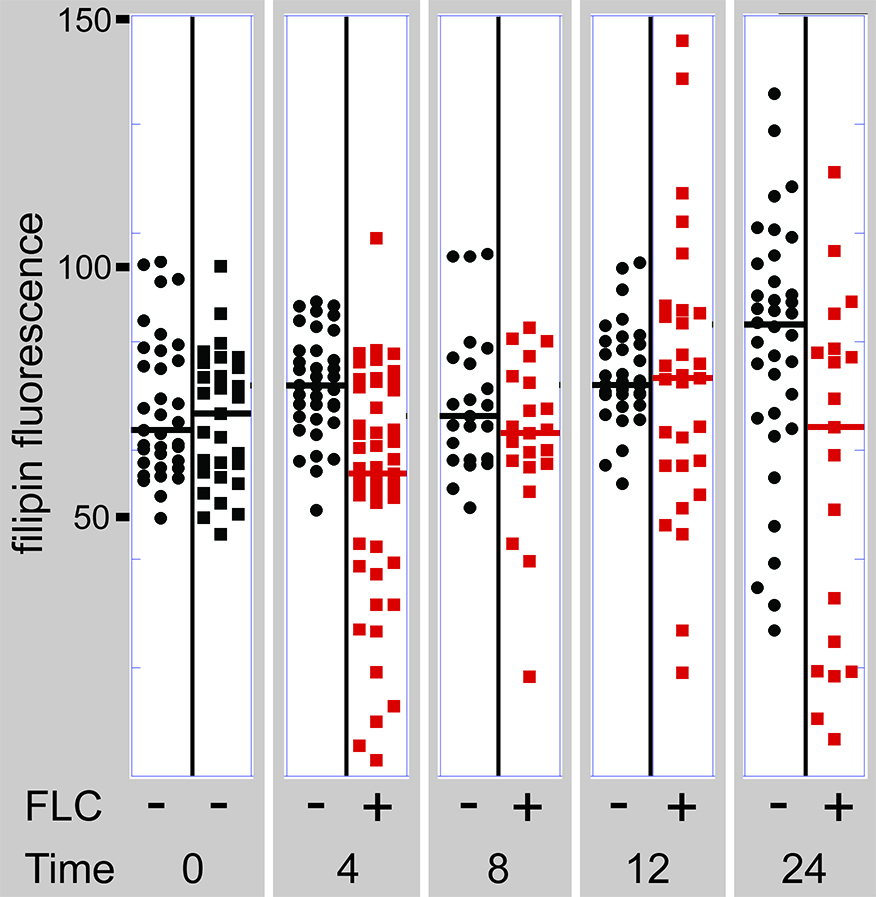

Supplement: Figure S1 — FLC treatment leads to unequal depletion of the plasma membrane ergosterol. The fluorescent dye filipin served as a proxy to examine the effect of FLC on the ergosterol content in the plasma membrane at a single cell level. A strain H99 was treated with 32 μg/ml FLC, whereas a strain expressing histone H4-mCherry was treated with DMSO and served as a control. This represents a reciprocal of the similar experiment depicted in Figure 1. The two strains were treated for indicated times. Prior to filipin staining strains were mixed in a 1:1 ratio, stained with filipin, and imaged immediately. The average filipin fluorescence of cells treated with FLC exhibited increase in variability compared to DMSO treated cells after 4 h of treatment. [file Image_1.JPEG]

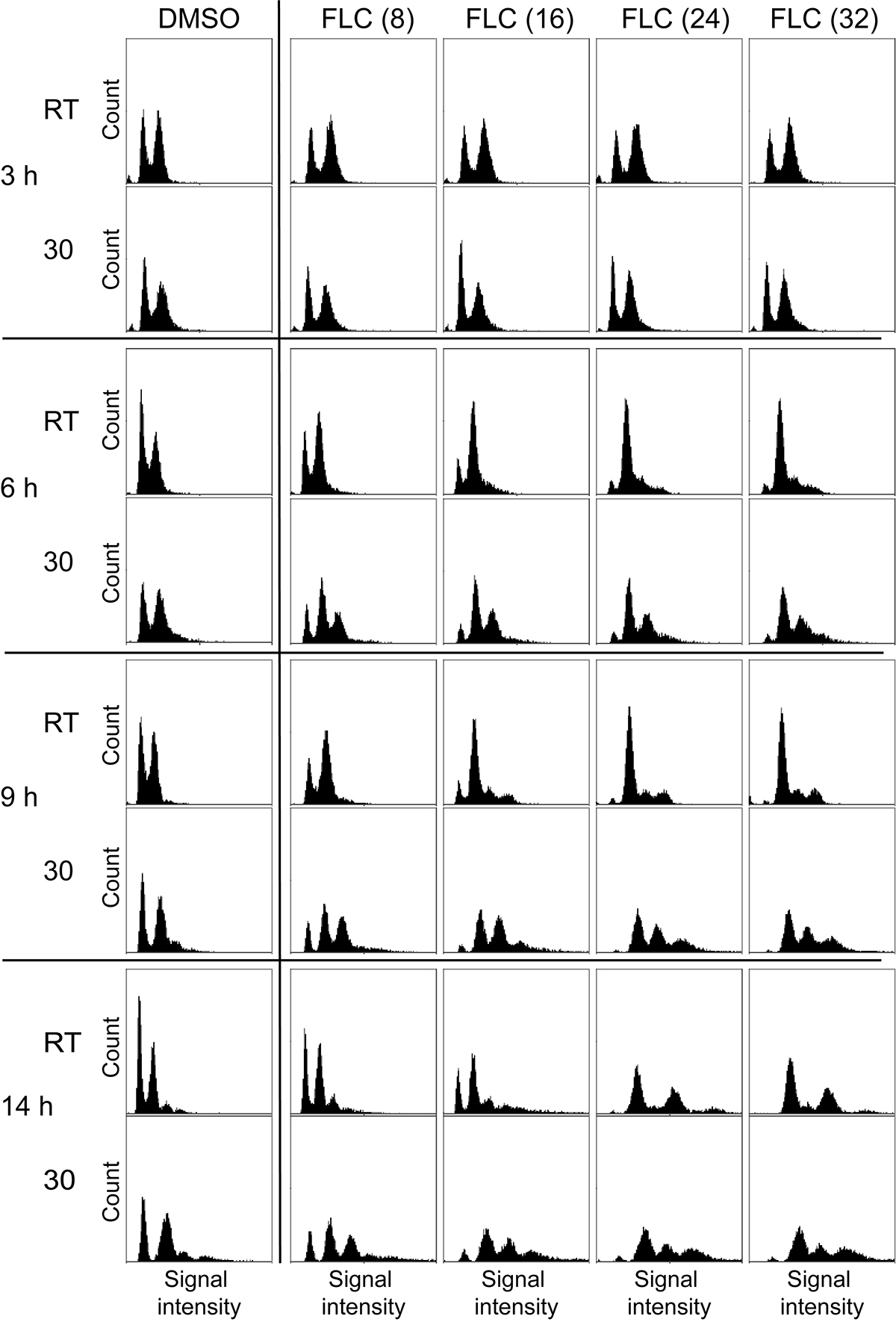

Supplement: Figure S2 — Increasing the incubation temperature leads to a more pronounced ploidy increase in FLC-treated cells. Cells were treated with DMSO (control) or FLC at indicated concentrations (μg/ml) for indicated times at either 24 (RT) or 30°C. Samples were fixed, stained with propidium iodide and analyzed by flow cytometry. [file Image_2.JPEG]

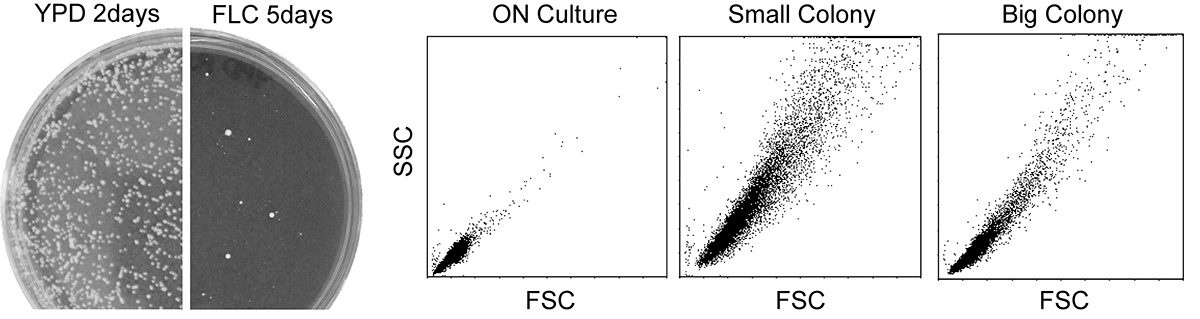

Supplement: Figure S3 — Analysis of the cell size and morphological complexity of cells. Cells were grown on YPD drug-free plates for 2 days or YPD plates supplemented with 32 μg/ml FLC for 5 days at 30°C. Cells from individual colonies were analyzed by flow cytometry to assess their sizes and morphological complexity. Cells obtained from the control YPD plate (ON culture) are uniform and relatively small. In contrast, cells obtained from the small colony (survivors) and the large colony (resistant cells) from the FLC-supplemented media show significant shift toward larger size and complexity. [file Image_3.JPEG]
